# Effectiveness of Topical Anesthetics in Pain Management for Dermal Injuries: A Systematic Review

**DOI:** 10.3390/jcm10112522

**Published:** 2021-06-07

**Authors:** Juan Manuel Navarro-Rodriguez, Carmen Suarez-Serrano, Rocio Martin-Valero, Yolanda Marcen-Roman, Maria de-la-Casa-Almeida

**Affiliations:** 1 Department of Nursing, University of Seville, 41009 Seville, Spain; jnavarro5@us.es; 2 Department of Physiotherapy, University of Seville, 41009 Seville, Spain; mcasa@us.es; 3 Department of Physiotherapy, University of Malaga, 29016 Malaga, Spain; rovalemas@uma.es; 4 Department of Nursing, Physiotherapy and Occupational Therapy, Institute for Health Research Aragón (IIS Aragón), University of Zaragoza, 50009 Zaragoza, Spain; yomarcen@unizar.es

**Keywords:** administration, topical, anesthetics, local, wounds and injuries

## Abstract

The treatment of dermal injuries is associated with pain in both adult and pediatric populations. We reviewed traditional treatments for controlling the pain of these lesions, such as infiltrated local anesthetics and topical local anesthetics. The objective of this review was to elucidate the efficacy of topical anesthetics in reducing the pain of dermal injuries, as well as the efficacy of topical anesthetics versus other anesthetics, or versus a placebo. Methodology: a systematic review was carried out by searching Medline (PubMED), Scopus, Cinahl, Cochrane, Lilacs, and ENFISPO for randomized clinical trials on the control of pain in dermal lesions through the use of topical anesthetics, versus a placebo or versus another anesthetic. Results: twelve randomized clinical trials with a total of 952 patients were included. Seven studies analyzed the efficacy of topical anesthetics compared to a placebo, and six of them observed statistically significant differences in favor of the experimental group. Five studies analyzed the efficacy of topical anesthetics compared to other anesthetics or sedatives; three of them observed statistically significant differences in favor of the experimental group, and two found no difference between the anesthetics analyzed. Conclusion: topical anesthesia is a useful method for pain control, is safe compared to other traditional methods, and offers a satisfactory form of pain relief in relation to infiltration anesthesia and compared to placebo.

## 1. Introduction

The treatment of dermal injuries is associated with pain in both adult and pediatric populations [1], and these lesions can occur in both acute and chronic states [2]. In both cases, pain control is vital [3], whether the pain occurs from the wound itself or is derived from the treatment administered.

For the control of pain in the curing of these lesions, we found sedation or anesthesia treatments, both at a local and general level [4]. As local anesthetics, infiltration and as well as topical anesthetics formulated in different presentation formats: liquid, gel, or patch, are found [2]. Despite the existence of these topical anesthetics, in the management of wounds for pain control, infiltrated anesthetics have traditionally been used [5], requiring a painful injection for anesthesia. However, in some cases, these can be avoided, or carried out without pain, by treating the area to be infiltrated using a topical anesthetic [6]. These topical anesthetics have been used as a treatment for the reduction of pain in wounds since the second half of the 19th century, with the discovery of cocaine [7]. A century later, many safer and more effective anesthetics are available as topical anesthetics [8]. In addition to reducing pain for the patient, topical anesthetics prevent distortion of the wound edge, which can be caused by subcutaneous infiltrates [9,10], as well as discomfort and anxiety caused by the infiltration itself.

Observing the benefits that topical anesthetics provide, both at the wound level and concerning the user’s well-being and safety, it is of special interest to know the efficacy of the different topical anesthetics used in the control of pain in the treatment of wounds as a treatment alternative to traditional infiltrated anesthetic treatment. Therefore, the objective of this systematic review is to explore the effectiveness of topical anesthetics in reducing pain from dermal injuries compared to other anesthetics or a placebo.

## 2. Methodology

### 2.1. Search Strategy

A systematic review was carried out on the efficacy of topical anesthetics in dermal injuries, according to the recommendations established for Systematic Reviews and Meta-analyzes (PRISMA) [11]. The review was registered in the PROSPERO database (CRD42020214902).

A search was carried out of the Medline (PubMED), Scopus, Cinahl, Cochrane, Lilacs, and ENFISPO databases.

For the search strategy, the following descriptors were selected from the Medical Subject Headings (MeSH): “administration, topical”, “anesthetics, local”, and “wounds and injuries”, and these from the Descriptors in Health Sciences (DeCS): “topical administration”, “local anesthetics”, “wounds and trauma”, and “injuries”.

The search strategy was as follows: (“administration, topical”) AND “anesthetics, local” AND (wounds OR injuries) for the Medline (PubMED), Scopus, Cinahl, and Cochrane databases, and (“topical administration”) AND “local anesthetics” AND (“wounds and traumatisms” OR “injuries”), for the databases in Spanish ENFISPO and Lilacs.

All included studies were carried out with humans, published between January 2005 and October 2020, and published in English, Italian, or Spanish.

### 2.2. Study Selection Criteria

The search was restricted to randomized clinical trials (RCTs). The articles that were included were those RCTs that compared the efficacy of a topical anesthetic with a placebo or another non-topical anesthetic in the treatment of dermal lesions, whose main variable was pain measured by any of the possible scales validated for this, without restriction regarding sex and age in the study population.

### 2.3. Study Variables/Outcomes

The main variable was pain, measured as pain intensity using one of any of the validated pain scales [12,13,14,15].

As a secondary variable, the existence or not of adverse effects was established, identifying which one(s) they were if any occurred.

As other variables to take into account, socio-demographic characteristics and information on the type of wound were collected.

### 2.4. Data Extraction

The main researcher carried out the search in the databases, and then made a first selection based on the title and the abstract, taking into account the established criteria. Subsequently, two independent researchers reviewed the full text of each of the selected studies, obtaining articles that integrated the results of this review. Observed discrepancies were resolved by consensus and, if this was unsuccessful, the participation of a third party was utilized.

These same two researchers independently extracted the qualitative and quantitative information of the main characteristics of the selected studies, as well as the results related to the main and secondary variables, summarizing them in tables made for this purpose. Once again, observed discrepancies were resolved by consensus and, if this was unsuccessful, through the participation of a third party.

Quality assessment of the selected articles was carried out by two independent researchers using the Jadad scale [16]. This scale measures the quality of clinical trials based on randomization, masking, and the description of possible losses to follow-up and dropouts from the study, with a range from 0 to 5, 0 being the lowest score and 5 the highest [16].

## 3. Results

### 3.1. Selection of Studies and Evaluation of Methodological Quality

**Figure 1:**
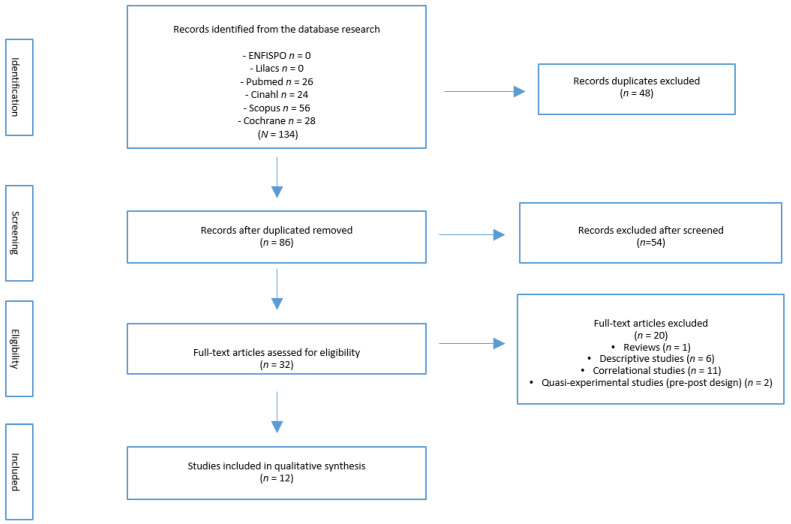
Flow diagram.

A total of 134 articles were identified from the various databases. After eliminating duplicates, a total of 86 remained, of which 32 articles were selected based on the title and abstract analysis. From the full text analysis of these 32 studies, according to the established selection criteria, a total of 12 RCTs were finally selected [17,18,19,20,21,22,23,24,25,26,27,28] (Figure 1).

The methodological quality of the studies, after applying the Jadad scale, is shown in Table 1; a mean score of 2.2 points was obtained.

### 3.2. Sociodemographic and Clinical Characteristics of the Population

A total of 952 subjects participated in the reviewed studies. A total of 719 adults were included in 10 of the 12 studies in our review, and 233 children, in the other two studies. The distribution by sex was 405 men and 470 women. The sample by sex does not coincide with the total number of participants, since the study by Gaufberg et al. [24] did not consider this variable.

Regarding the location of the wounds, one study analyzed the use of topical anesthetics in corneal injuries [20], one in the knee [19], two in the oral area, two in the perianal area [17,23], one in lower limbs exclusively [21], and four in wounds located on different parts of the body, such as the shoulder, ankle, knee, lower limbs, upper limbs, face, torso or hands [18,24,25,26]. The final study was on burns, without reference to their location but rather their extension only, which was between 1% and 5% of the total body surface [27] (Table 2).

### 3.3. Characteristics of the Intervention

Regarding the type of intervention, four of the studies analyzed the efficacy of topical anesthetics in wound suturing [17,23,25,26], one in the removal of metallic sutures [19], and one in the control of pain caused by the wound itself [20]. Five studies analyzed the anesthetic effect during the development of cures [21,22,24,27,28], and one during vacuum withdrawal in lower limb ulcers [18] (Table 2).

Regarding the comparison of the interventions, it should be noted that, of the 12 studies, 7 analyzed the efficacy of a topical anesthetic versus a placebo (Table 3) [18,19,20,22,25,27,28], and 5 compared a topical anesthetic with another type of anesthetic or sedative (Table 4) [17,21,23,24,26].

In relation to the topical anesthetics used, we observed great variability. Depending on the type of presentation, they could be in a liquid format [18,20,24], cream [17,21,22,23,27], emulsion [25,28], or dressing [19,26]. The concentrations of the active principle ranged from 1% [18,20,22] to 5% [19,24], including concentrations of 2% [28], 2.5% [17,21,23,27], and 4% [26]. The active ingredients included lidocaine [17,18,19,21,22,23,24,25,26,27], prilocaine [17,21,23,27], and tetracaine [20,25]. Based on their composition, topical anesthetics with unique active principles were found [18,19,20,22,26,28], along with combinations of various anesthetics (lidocaine/prilocaine) [17,21,23,27], or anesthetics combined with vasoconstrictor medications [24,25].

Other characteristics related to the type of anesthetic of the experimental group and/or placebo used in each study are shown in Table 2.

### 3.4. Main Variable: Pain

Of the analyzed studies, 11 used the “Visual Analogic Scale” (VAS) to measure pain [17,18,19,20,21,22,23,24,25,26,28], one used the “Numerical Rating Scale” (NRS) [27], one used the “Face Scale of Pain” (FSP) [25], and one used the “Verbal Rating Scale” (VRS) [21] (Table 2). Two of the 12 studies used two scales to measure pain; Claeys et al. [21] used the VAS scale and the VRS scale with all their patients; Harman et al. [25] used the VAS and FSP, depending on the age of the study subjects (those under seven years old used the FSP scale and those seven years or older the VAS scale).

Topical vs. placebo effectiveness: among the seven studies that analyzed the efficacy of a topical anesthetic versus a placebo (Table 3), six of them observed statistically significant differences in favor of the experimental group [18,19,22,25,27,28]. On the other hand, in their study, Waldman et al. [20] did not observe differences between the two groups.

Christensen et al. [18] observed a difference in pain during the removal of a vacuum dressing (VAC) between both groups and, after 5, 10, and 20 min, found a statistically significant difference in all the outcomes (*p* < 0.001, *p* = 0.001, *p* = 0.036, and *p* = 0.004, respectively). Descroix et al. [22] observed a statistically significant difference between both groups in the pain associated with periodontal wounds at three minutes after the application of the anesthetic, using the VAS scale (*p* = 0.0001), with no significant difference between groups during the application of the anesthetic (*p* = 0.22). During the application of adhesive sutures, Harman et al. [27] observed a statistically significant decrease in pain, based on both scales used in their study, in favor of the experimental group (VAS scale and FSP scale (*p* = 0.01 and *p* < 0.01, respectively)). After the healing of burns, Kargi et al. [27] measured pain using the VAS scale across three periods of 8 h, observing statistically significant differences (*p* < 0.05) between groups in favor of the topical anesthetic in the first and second periods of 8 h, but not observing a statistically significant difference in the third period of 8 h after the application of the anesthetic. Kasaj et al. [28] did not observe a statistically significant difference (*p* > 0.05) immediately after periodontitis scaling in their groups; However, they observed a statistically significant difference in both groups (*p* < 0.001), and in favor of the experimental group over the control (*p* < 0.001) at 10, 20, and 30 min after the application of the anesthetic. During staple removal from surgical wounds, Tseng et al. [19] observed a statistically significant difference between groups in favor of the experimental group, both on the VAS scale (*p* = 0.001) and on the FPS scale (*p* < 0.001).

On the other hand, Waldman et al. [20], in corneal lesion treatment, did not observe statistically significant differences between the anesthetic group and the placebo when measuring pain after the first 24 h (*p* = 0.259), and then 48 h (*p* = 0.149), after application of the topical anesthetic every two hours.

Topical effectiveness vs. other anesthetics: regarding the five studies that analyzed the efficacy of a topical anesthetic against other anesthetics or sedatives (Table 4), three of them observed statistically significant differences in favor of the experimental group [17,21,23], and two did not observe differences between groups [24,26].

Claeys et al. [21], during the debridement of ulcers, observed a statistically significant difference in favor of the topical anesthetic versus the inhaled sedative, as measured by the VAS scale (*p* < 0.001) and VRS scale (*p* < 0.001). Franchi et al. [23], similarly, observed during episiotomy, and measuring pain with the VAS scale, a statistically significant difference between groups (*p* = 0.002) in favor of the experimental group. Abbas et al. showed a statistically significant difference between groups (*p* = 0.001) in favor of the experimental group [17] when measuring pain with the VAS scale during episiotomy.

On the other hand, Gaufberg et al. [24] did not observe significant differences between two types of anesthetics, topical and infiltrate, which were equally effective in both groups during suturing (*p* = 0.59). The results observed by Jenkins et al. [26] on the use of topical anesthetic versus infiltrate were similar; they did not observe a statistically significant difference between the groups during wound suturing.

### 3.5. Secondary Variable: Adverse Effects

Adverse effects were evaluated in 6 of the 12 studies analyzed [17,18,19,24,26,28] (Table 2), and were found in only two of them [17,26] (Table 3 and Table 4).

In the studies that analyzed topical anesthetics versus placebo, no adverse effects were identified.

Among the studies that analyzed the efficacy of a topical anesthetic versus another anesthetic, only two described any adverse effects. Abbas et al. [17] observed, in the topical anesthetic group, a tingling sensation in two patients (2.8%) and a burning sensation in one (1.4%). In the infiltrated anesthetic group, the sensation of swelling in the suture area was described by two patients (2.8%). Jenkins et al. [26] observed wound infection in 3.7% of the patients studied in the topical anesthetic group, suture dehiscence in 3.7%, and the need for re-suturing in 1.8%, while, in the infiltrated anesthetic group, they observed infection in 7.1% of the patients, and the need for re-suturing in 1.8%, with no suture dehiscence appearing in this group (Table 3).

## 4. Discussion

The results discussed here show that the administration of topical anesthetics is effective and safe for the management of pain in injuries at the dermal level, even in mucous membranes. In 11 of the 12 clinical trials reviewed, topical anesthetics were found to be efficacious for pain management. Only in Walman et al.’s study [20] did the authors not observe differences between groups in the control of pain in corneal lesions, which could be related to timing of the pain evaluations. In this study, these were undertaken two hours after each application of the topical anesthetic, which may have been sufficient time for the anesthetic effect to wear off, or the pain level may have been influenced by the anatomophysiology itself.

Despite the fact that the reviewed studies were clinical trials, the mean obtained by the Jadad scale was 2.2 points out of a maximum of 5, which implies a low methodological quality, according to Jadad et al. [29]. Ten of the trials included in our review obtained a score of 3 or less, which suggests serious biases in the trials, and therefore that they may be studies of low methodological quality [29]. Only two of the trials showed acceptable methodological quality, with a score of 4 on the Jadad scale [29]. In these studies, the efficacy of a topical anesthetic was evaluated against a placebo, with very different results in each. This indicates a need for studies that unify the type of lesion and the type of anesthetic, as well as the different variables to be studied, and adapt both the masking and the randomization in order to evaluate these types of anesthetic treatments. Descroix et al. [22] supported the efficacy of pain control achieved by topical anesthetics in oral lesions, and Waldman et al. [20] concluded that there were no significant differences between the group that received a topical anesthetic and the group that received saline (placebo) in the management of corneal lesions. These differences could be due to the measurement times and the duration of the anesthetic effect. According to Stern and Giddon [30], if a topical anesthetic is applied to the oral mucosa, analgesia of the application area is achieved for up to 21 min after its application. Descroix et al. [22] described the efficacy of the anesthetic three minutes after its application. Kargi and collaborators [27] studied the effect in the first 24 h, and noted a reduction in the anesthetic effect in burns in the last 8 h of the day, after the anesthetic was initially effective in the first 16 h. However, in the study by Waldman et al. [20], the effect had possibly disappeared when the pain measurements were taken after two hours.

Regarding the concentration of the active principle, in 11 of the 12 trials, using concentrations from 1% to 5%, effectiveness in controlling pain was achieved with all three forms of anesthetic presentation—liquid [18,24,25,26,28], cream [17,21,22,23,27], or patch [19]—during the debridement of ulcers in the lower limbs [21], in the control of pain in canker sores [22], during suturing in episiotomies [17,23], during burn healing [27], and as an alternative to infiltrated anesthesia in wound closure [24,25,26]. Waldman et al. [20] used 1% tetracaine formulated as drops for the treatment of pain in ocular ulcers, concluding its ineffectiveness. The results observed by Waldman et al. [20] may be due to the physiology of the eye itself, wherein the epithelia of the ocular surface are unable to adequately absorb the medication, or to the possible losses generated by the systemic absorption of the medication through the conjunctiva and the nasolacrimal duct. Another difficulty in exploring the efficacy of anesthetics with respect to their concentration could be due to the different medications reviewed, since Waldman et al. were the only authors to use unmixed tetracaine as a topical anesthetic.

In trials where topical anesthetics other than tetracaine were used, alone or in combination, their efficacy in pain control was confirmed. EMLA^®^ (Aspen Pharma Trading Limited, Dublin, Ireland) cream, which is 2.5% lidocaine with 2.5% prilocaine, was used successfully in several trials [17,21,23,27]. The combination of two anesthetics, such as lidocaine with tetracaine, and a vasoconstrictor was utilized in several studies [25,31], and this also appeared to be an effective formula for pain control.

We found important differences in the results in the effect of the topical anesthetics over time. In the study carried out by Christensen and collaborators [18], the effect of the anesthetic, in liquid form, for pain control in mouth ulcers, was maintained during the first 20 min, with a difference of 2 points with respect to the control group. In its gel presentation, it was effective for the first 30 min [28]. On the contrary, Desai and collaborators [32] observed that the liquid formula showed a reduced effectiveness of the anesthetic after 60 min, while the cream was more effective after the first 60 min. Waldman et al. [20] did not find any anesthetic effect of a liquid formula when it was applied to corneal lesions, which could have been due to the physiology of the cornea, as discussed above.

Regarding the exposure or administration times, although Patterson, in 1998 [33], reported that the exposure time for topical anesthetics should be 20–30 min for LAT and between 30 min and 2 h for EMLA^®^ cream, recent studies have suggested the need for a shorter exposure time to achieve anesthesia, with exposure times ranging from 5 or 15 min [34] to 20 min [35] for the gel LAT, and 45 min, 60 min [17], or up to 2 h [36] for EMLA^®^ cream.

In our review, we found that Descroix et al. [22] observed a decrease in pain three minutes after the application of EMLA^®^ cream to the oral mucosa. Kargi and collaborators [27], during the treatment of burns, showed effectiveness in the first 8 and 16 h after its application. According to these results, the area to which the topical anesthetics are applied can influence the duration of pain.

Another aspect to assess in the use of topical anesthetics is the plasma concentration of the anesthetic in the blood after application. According to Desai et al. [32], the exposure time does not influence this concentration: after one hour in contact with the anesthetic, they observed that the plasma lidocaine concentrations in the blood were significantly lower (25 times less for treatment with 4% anesthetic, and 70 times less for treatment with 3% anesthetic) than the recommended maximum toxic plasma concentration of >5 mg/l [5]. The study by Kargi et al. [27] also found that the concentrations of lidocaine and prilocaine were below toxic levels after 4 and 8 h of contact with the topical anesthetic.

Considering the results obtained in our review, topical anesthetics appear to be an effective form of anesthesia in dermal injuries, since they have been shown to be effective compared to placebo treatments, as well as more effective than other non-infiltrated anesthetics, and just as effective in two of the three studies that analyzed their efficacy against infiltrates. Due to their preferable application method, they could provide a greater degree of satisfaction and comfort to both the patient and the healthcare personnel who administers it, especially when the observed adverse effects are minimal. In addition, Franchi et al. [23] and Gaufberg et al. [24] observed that patients treated with topical anesthetics had a satisfactory experience during their application, unlike to the infiltrated anesthetic. The degree of safety was assessed in the study carried out in 2013 by Harman et al. [25], where they analyzed the difficulty of suturing wounds, and observed a greater difficulty in wound closure in the control group. This difficulty was caused by bleeding from the wound itself. Thus, the topical anesthetic also improved the control of bleeding in the wounds, improving safety in the treatment of bleeding wounds [31].

Patient perception is essential for the acceptance of pain control in anesthetic treatment, especially in children [37]. The satisfaction expressed by the patient was analyzed in four studies and, in one, the satisfaction of the healthcare personnel was also analyzed [17,23,24,25]. In the studies where the effect of the topical anesthetic versus an infiltrated anesthetic was analyzed, different trends appeared with respect to satisfaction with the pain control treatment between groups. Franchi et al. [23] observed greater satisfaction during episiotomy suturing in the experimental group compared to the control group (83.8% vs. 53.3%), as did Abbas et al. [17] and Gaufberg et al. [24].

In the reviewed studies, where the inclusion of adjuvant rescue medication for pain control in the anesthetic treatment was analyzed, reduced use of rescue medication was observed in the experimental groups, which demonstrates the efficacy of the anesthetic. We found correlations in the lower use of analgesic medication in the groups with the use of topical anesthesia [18,27], and a similar use with respect to traditional methods [23].

Regarding the appearance of side effects associated with any anesthetic method, in four of the seven studies that analyzed adverse effects, no adverse effects were detected [18,19,24,28] and, in those where adverse effects occurred, they were present in both groups. Two [17,26] assessed the same type of intervention, suturing, which could suggest that the adverse events were related more to the intervention than to the anesthetic, although in the study by Gaufberg et al. [24], where wound suturing was also carried out, no adverse effects were observed in any of the groups. The adverse effects observed in the study by Waldman et al. [20] could have been related to the idiosyncrasies of ocular lesions and not to the anesthetic, since the observed effects appeared in the same way and form in both groups.

Six of the seven studies comparing topical anesthetics with a placebo agreed on the greater anesthetic efficacy of the topical, regardless of the procedure. We can conclude that the effectiveness of topical anesthetics when used for injuries is clear since, in all the included trials, significantly positive differences in favor of the infiltrate were not observed.

The five studies that compared topical anesthetics with other anesthetics showed that topical anesthesia can be an effective alternative to infiltrated anesthesia [17,23,24,26] in procedures such as wound suturing, or to nitrous oxide analgesia [21] in the debridement of wounds in the lower limbs.

## 5. Limitations

Few studies have investigated the anesthetic effect of topically applied medication in the treatment of dermal lesions, and the vast majority of existing studies have shown great variability in the measurements collected and are of low methodological quality. Studies with higher methodological quality are required in order to clarify the efficacy of topical anesthetics for pain control and, thus, perform a quantitative analysis on this topic.

Due to the heterogeneity of the studies included in terms of the lesions, measurements tools, measurements times, and the type and dose of topical anesthetic used, in addition to the great variability in the presentation of the results of these studies, it has not been possible to carry out a meta-analysis on this subject.

## 6. Conclusions

Topical anesthesia is a useful method for pain control and is safe compared to other traditional methods, offering a satisfactory form of pain relief when compared to infiltration anesthesia and placebo.

However, further research is needed with good methodological quality to demonstrate the effectiveness of this type of anesthetic treatments in dermal injuries.

## Figures and Tables

**Table 1:** Quality assessment of the included studies (Jadad score).

Authors & Year	Was the Study Described as Randomized?	Was the Method of Randomization Appropriate?	Was the Study Described as Blinded?	Was the Method of Blinding Appropriate?	Was there a Description of Withdrawals and Dropouts?	Total
Abbas et al., 2019 [17]	1	1	0	0	0	2
Christensen et al., 2013 [18]	1	0	1	0	0	2
Claeys et al., 2010 [21]	1	1	0	0	0	2
Descroix et al., 2011 [22]	1	1	1	1	0	4
Franchi et al., 2009 [23]	1	0	0	0	0	1
Gaufberg et al., 2006 [24]	1	0	0	0	0	1
Harman et al., 2013 [25]	1	1	1	0	0	3
Jenkins, 2010 [26]	1	1	0	0	0	2
Kargi et al., 2010 [27]	1	0	0	0	0	1
Kasaj et al., 2007 [28]	1	0	1	0	0	2
Tseng et al., 2017 [19]	1	0	1	0	0	2
Waldman et al., 2014 [20]	1	1	1	1	0	4

**Table 2:** Characteristics of the included studies.

Author and Year	Sample Size EG/CG	Intervention	Wound or Injury	Intervention		Outcomes	
						Primary	Secondary
				Intervention 1 (EG)	Intervention 2 (CG)	Pain	Adverse Effects
Abbas et al., 2019 [17]	*n* = 14	Suture	Perineal tears	Lidocaine (25 mg/g) with prilocaine (25 mg/g)	mepivacaine infiltrated	Pain (VAS)	Evaluate
	72/72						
Christensen et al., 2013 [18]	*n* = 11	Removal of wound Vacuum-Assisted Clousure dressing (VAC)	Venous leg ulcers	Lidocaine 1%	Normal saline	Pain (VAS)	Evaluate
	11/11						
Claeys et al., 2010 [21]	*n* = 41	Debridement	Chronic arterial and venous leg ulcers	Lidocaine (25 mg/g) with prilocaine (25 mg/g)	Nitrous oxide oxygen mixture inhalated 9−12 L/min (max. 30 min)	Pain (VAS)	No Evaluate
	20/21					Verbal Rating Scale (VRS)	
Descroix et al., 2011 [22]	*n* = 59	wound management	Oral mucosal trauma or oral aphthous ulcer	Lidocaína crema 1% Dosis 0.2 g	placebo cream	Pain (VAS)	No Evaluate
	29/30						
Franchi et al., 2009 [23]	*n* = 61	Suture	Perineal tears	Lidocaine (25 mg/g) with prilocaine (25 mg/g)	mepivacaine infiltrated	Pain (VAS)	No Evaluate
	31/30						
Gaufberg et al., 2006 [24]	*n* = 100	wound management	Wounds	Topical lidocaine 5% with epinephrine 0.025%	lidocaine infiltrated 2%	Pain (VAS) during repair of wound	Evaluate
	50/50						
Harman et al., 2013 [25]	*n* = 203	Suture	Wounds	LET (lidocaine, epinephrine, tetracaine)	placebo	Pain (VAS)	No Evaluate
	105/98					Face Scale of Pain (FSP)	
Jenkins, 2010 [26]	*n* = 110	Suture	Wounds	Topical lidocaine 4%	Lidocaine infiltrated 1%	Pain (VAS)	Evaluate: Wound infecction
	56/54						Wound dehiscence
							Resuture
Kargi et al., 2010 [27]	*n* = 30	wound management	Burns	Lidocaine-prilocaine 5%	antibiotic cream	verbal Numerical Rating Scale (NRS)	No Evaluate
	15/15						
Kasaj et al., 2007 [28]	*n* = 40	wound management	Chronic periodontitis	Lidocaine 20 mg/g gel	placebo	Pain (VAS)	Evaluate
	20/20						
Tseng et al., 2017 [19]	*n* = 60	Staple removal	Surgical wounds on the knee	Lidocaine patch 5% (700 mg patch)	Placebo patch	Pain (VAS)	Evaluate: skin allergy wound infection
	30/30						
Waldman et al., 2014 [20]	*n* = 93	Pain management	Corneal abrasions	Tetracaine 1%	Normal saline	Pain (VAS)	Evaluate
	46/47						

EG, experimental group; CG, control group; VAC, vacuum assisted closure; LET, lidocaine epinephrine tetracaine; VAS, visual analogue scale; VRS, verbal rating scale; FSP, face scale of pain; NRS, verbal numerical rating scale.

**Table 3:** Results topical anesthetic vs. Placebo (^†^ median).

KERRYPNX	Intervention		Outcomes			
			Primary		Secondary	
			Pain		Adverse Effects	
Author and Year	Intervention 1 (EG)	Intervention 2 (CG)	EG	CG	EG	CG
Christensen et al., 2013 [18]	Lidocaine 1%	Normal saline	During remove (−2.4) *p* < 0.001.		Not evaluate	
			5 min: (−2) *p*: 0.001.			
			10 min: (−1.5) *p*: 0.036.			
			20 min: (−1.8) *p*: 0.004			
Descroix et al., 2011 [22]	Lidocaine cream 1%	Placebo cream	VAS: T0: 55.3 (±8.4)	VAS: T0: 57.2 (±10.6)	Not evaluate	
			VAS: T3: 25.9 (±17.6)	VAS: T3: 42.5 (±15.5)		
Harman et al., 2013 [25]	LET (lidocaine, epinephrine, tetracaine)	Placebo	VAS: 0.5 ^†^	VAS: 1 ^†^	Not evaluate	
			FSP: 0 ^†^	FSP: 2 ^†^		
Kasaj et al., 2007 [28]	Lidocaine 20 mg/g gel	Placebo	VAS: during: 5.2 (±1.4)	VAS: during: 5.5 (±1.4)	No adverse effects	
			VAS: 10 min: 0.3 (±0.1)	VAS: 10 min: 3.2 (±1.9)		
			VAS: 20 min: 0.3	VAS: 20 min: 2.1 (±1.6)		
			VAS: 30 min: 0.3	VAS: 30 min: 1.7 (±1)		
Tseng et al., 2017 [19]	Lidocaine patch 5% (700 mg patch)	Placebo patch	VAS: 4.7 (±1.6)	VAS: 6.5 (±1.9)	No adverse effects	
Waldman et al., 2014 [20]	Tetracaine 1%	Normal saline	VAS: baseline 5.46	VAS: baseline: 4.8	Retained rust rings: 22%.	Retained rust rings: 17.5%, *p* = 0.544)
					Flurescein uptake: 23.9%	Flurescein uptake: 21.3%, *p* = 0.761
					Persitent symptoms: 21.7%	Persitent symptoms: 21.3%, *p* = 0.957)
			VAS: 24 h: difference 0.44	VAS: 24 h: difference 0.44	Persistent symptoms after a week: 2.2%	Persistent symptoms after a week: 8.5%, *p* = 0.176.
			VAS: 48 h: difference 0.53	VAS: 48 h: difference 0.53		

EG = experimental group; CG = control group; LET = lidocaine epinephrine tetracaine; VAS = visual analogue scale; FSP = face scale of pain.

**Table 4:** Results of topical anesthetic Vs. Non topical anesthetic.

Author and Year	Intervention		Outcomes			
			Primary		Secondary	
			Pain		Adverse Effects	
	Intervention 1 (EG)	Intervention 2 (CG)	EG	CG	EG	CG
Abbas et al., 2019 [17]	Lidocaine (25 mg/g) with prilocaine (25 mg/g)	mepivacaine infiltrated 2%	VAS: 3.86 (±1.59)	VAS: 5.99 (±1.47)	Tingling: 2.8%	Tingling: 0%
					Swelling: 0%	Swelling: 2.8%
					Burning sensation: 1.4%	Burning sensation: 0%
Claeys et al., 2010 [21]	Lidocaine (25 mg/g) with prilocaine (25 mg/g)	Nitrous oxide oxygen mixture inhalated 9−12 l/min (max. 30 min)	VAS: 3.68 (±0.25)	VAS: 5.29 (±0.27)	Not evaluate	
			VRS: 1.71 (±0.15)	VRS: 2.87 (±0.15)	Not evaluate	
Franchi et al., 2009 [23]	Lidocaine (25 mg/g) with prilocaine (25 mg/g)	mepivacaine infiltrated 1%	VAS: 1.7 (±2.4)	VAS: 3.9 (±2.4)	Not evaluate	
Gaufberg et al., 2006 [24]	Topical lidocaine 5% with con epinephrine 0.025%	lidocaine infiltrated 2%	VAS: 0.16 (±0.46)	VAS: 0.2 (±0.49)	No adverse effects	
Jenkins, 2010 [26]	Topical lidocaine 4%	lidocaine infiltrated 1%	VAS: 1.49 (±1.76)	VAS: 0.78 (±1.12)	Wound infection: 3.7% Wound	Wound infection: 7.1% Wound
					3.7% Deshiscence	0% Deshiscence
					1.8% Resuture	1.8% Resuture

EG = experimental group; CG = control group; VAS = visual analogue scale; VRS = verbal rating scale.
